# Identification and quantification of selected metabolites in differently pigmented leaves of lettuce (*Lactuca sativa* L.) cultivars harvested at mature and bolting stages

**DOI:** 10.1186/s13065-019-0570-2

**Published:** 2019-04-19

**Authors:** Awraris Derbie Assefa, Susanna Choi, Jae-Eun Lee, Jung-Sook Sung, On-Sook Hur, Na-Young Ro, Ho-Sun Lee, Suk-Woo Jang, Ju-Hee Rhee

**Affiliations:** 1 National Agrobiodiversity Center, National Institute of Agricultural Sciences, RDA, Jeonju, 54874 South Korea; 2 Vegetable Research Division, National Institute of Horticultural & Herbal Science, RDA, Wanju, 55365 South Korea

**Keywords:** Lettuce, ABTS·^+^, Anthocyanins, Sesquiterpene lactones, Phenolic acids, Flavonoids

## Abstract

**Background:**

Identification and screening of cultivars rich in bioactive phytoconstituents can be potentially useful to make nutrient-dense dishes and in medicinal formulations. In this study, we have identified, characterized and quantified caffeoylquinic acids, dicaffeoylquinic acid, dicaffeoyltartaric acid, kaempferol conjugates, quercetin malonylglucoside, sesquiterpene lactones, and cyanidin in 22 lettuce cultivars at mature and bolting stages using UPLC-PDA-Q-TOF-HDMS, UPLC, and HPLC.

**Results:**

The composition and contents of the studied metabolites and antioxidant activity varied significantly and depend on leaf color, cultivar type and stage of maturity. The main phenolic acid components of lettuce were quinic and tartaric acid derivatives, whereas kaempferol derivatives were the dominant flavonoids. The sum of the content of phenolic acids ranged from 18.3 to 54.6 mg/100 g DW and 15.5 to 54.6 mg/100 g DW, whereas the sum of the contents of flavonoids ranged from 9.2 to 25.9 mg/100 g DW and 14.9 to 83.0 mg/100 g DW in mature and bolting stage cultivars, respectively. The content of cyanidin, lactucin, lactucopicrin, and ABTS radical antioxidant activity were in the range of 0.3 to 9.7 (mature stage) and 0.5 to 10.2 mg/g DW (bolting stage), 1.8 to 41.9 (mature stage) and 9.7 to 213.0 (bolting stage) µg/g DW, 9.9 to 344.8 (mature stage) and 169.2 to 3888.2 (bolting stage) µg/g DW, and 12.1 to 29.0 (mature stage) and 15.7 to 30.3 (bolting stage) mg TE/g DW, respectively. The principal component analysis (PCA) showed that the green and red pigmented lettuce cultivars were grouped to the negative and positive sides of PC1, respectively, while the green/red pigmented cultivars were distributed throughout the four quadrants of the PCA plots with no prominent grouping. The loading plot showed that phenolic acids, flavonoids, and cyanidin are the most potent contributors to the radical scavenging activity of lettuce extracts.

**Conclusions:**

Lettuce at the bolting stage accumulate relatively high amount of sesquiterpene lactones (SLs), quercetin malonylglucoside (QMG), methylkaempferol glucuronide (MKGR), kaempferol malonylglucoside (KMG), and 3-*O*-caffeoylquinic acid (3-CQA) compared to the mature stage. Higher amount of phytoconstituents were found to be accumulated in the red pigmented lettuce leaves compared to the green lettuce leaves. In addition, the contents of most of the metabolites in lettuce seem to increase with age of the leaves. The presence of the two bitter SLs, lactucin and lactucopicrin, in significantly high amount in lettuce leaves at bolting stage could diminish consumer acceptance. However, alternatively, these leaves could be utilized by nutraceutical companies working to recover these compounds.
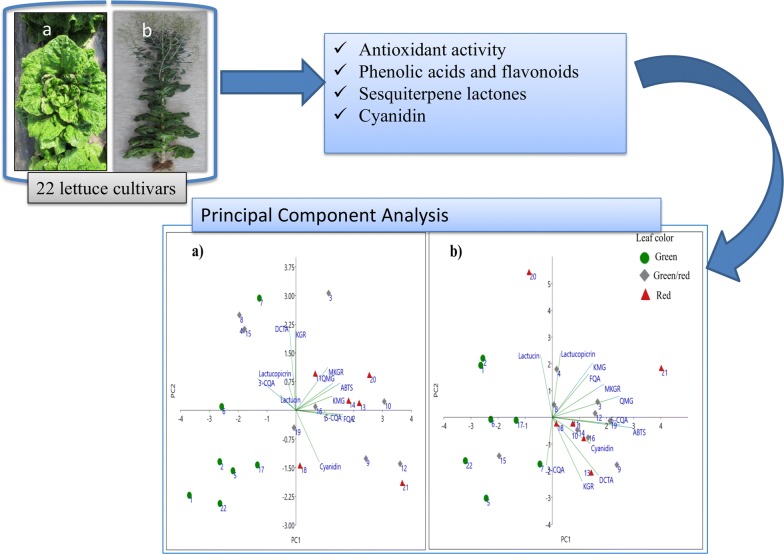

**Electronic supplementary material:**

The online version of this article (10.1186/s13065-019-0570-2) contains supplementary material, which is available to authorized users.

## Introduction

Lettuce, one of the most popular vegetables in terms of production and economic value, ranks second to potato in per capita consumption [1]. It is planted annually in backyards, containers, shade net, and greenhouses even through hydroponics with a range of environmental conditions. Lettuce is an important crop because of its increased productivity per unit area of cultivated land and its suitability in vertical farming systems [2]. Lettuce cultivar type could be classified based on head shape such as crisphead, butterhead, cos (romaine), and leafy. It is usually grown for its leaf, but sometimes for its stem and seeds. In Korea, lettuce is mainly consumed as a salad dish or eaten with sandwiches and meat [3]. In recent years, consumers’ interest in lettuce has increased dramatically due to its attractive visual quality, minimum microbial load, and presence of nutritionally important phytoconstituents [1]. Moreover, lettuce contains several bioactive phytochemicals including, anthocyanins [4], phenolic acids [5, 6], flavonoids [5, 7], carotenoids, folate, ascorbic acid [8], and sesquiterpene lactones [3, 9]. In addition, lettuce plants did show potent allelopathic activity [10].

The composition of phytochemicals in lettuce could be affected by several factors such as genetic makeup, mulching, and storage conditions [11, 12], planting date [4, 13], temperature [14], processing [12], leaf position, and head formation stage [15], type (leaf/head/romaine) [16], and harvesting stage [11, 17]. On the other hand, allelopathic effects of lettuce which could be due to their sesquiterpene lactones content vary depending on the type of cultivar [10]. Lettuce leaf color is an important factor not only because it is the first trait that registers with consumers/buyers but also it indicates the presence/absence of some beneficial metabolites to the plant and human health [1, 6]. Obviously, lettuce at the bolting stage is more exposed to environmental conditions (nutrient absorptions, temperature, humidity, and light) compared to mature stage due to a longer period of cultivation. Hence, one would expect a biochemical conversion, degradation, or accumulation throughout the growing season.

Lettuce goes through distinct growth stages including, emergence of cotyledons leaves, formation of distinct circular cluster of leaves (rosette stage), inward curling of tips of inner leaves (cupping stage), overlapping of the cupped leaves and covering the growing point of the plant (heading stage), maturing where head/leaf reaches marketable size, and finally bolting where the main shoot inside head begins to elongate. To date, studies on the lettuce composition of phenolic compounds and its potential as antioxidant were focused on baby-leaf and mature stages of lettuce [1, 8, 18, 19]. For example, Kim et al. [1] studied the carotenoids, cyanidin, fatty acids, and total folate profiles of 23 cultivars of baby-leaf lettuce only. Studies on the composition of sesquiterpene lactones and anthocyanin content in the leaves of lettuce at different stages of various types of lettuce are elusive. Thus, in this study, the profiles of phenolic acids, flavonoids, anthocyanidins, sesquiterpene lactones and antioxidant activity in leaves of lettuce cultivars at the mature and bolting stages of lettuce were examined. Also, it is discussed how the lettuce phytochemicals are affected by genotype, leaf color, and maturity. The obtained quantitative data were analyzed using principal component analysis (PCA) to distinguish the studied cultivars based on their leaf color. The knowledge on the phytochemicals distribution in differently pigmented lettuce cultivars at mature and bolting stages could be of interest to consumers and food industry especially in selecting suitable cultivar and stage of development to make lettuce related nutrient-dense dishes and to extract health promoting phytochemicals that could potentially be useful in medicinal formulations.

## Materials and methods

### Chemicals, reagents, and standards

All chemicals and solvents used in extraction and analysis were of analytical grade and purchased from Fisher Scientific Korea Ltd. (Seoul, South Korea) and Sigma-Aldrich (St. Louis, MO, USA). Standards lactucin, lactucopicrin, kuromanin chloride (cyanidin 3-*O*-glucoside chloride) were HPLC grade (> 95% purity) and were purchased from Extrasynthese (Lyon, France). Other standards such as 5-*O*-caffeoylquinic acid (5-CQA), dicaffeoyltartaric acid (DCTA), quercetin 3-*O*-(6″-malonyl)-β-d-glucoside (QMG), and kaempferol 3-*O*-glucoside (KG) were purchased from Sigma-Aldrich (Seoul, Korea).

### Plant material

**Fig. 1:**
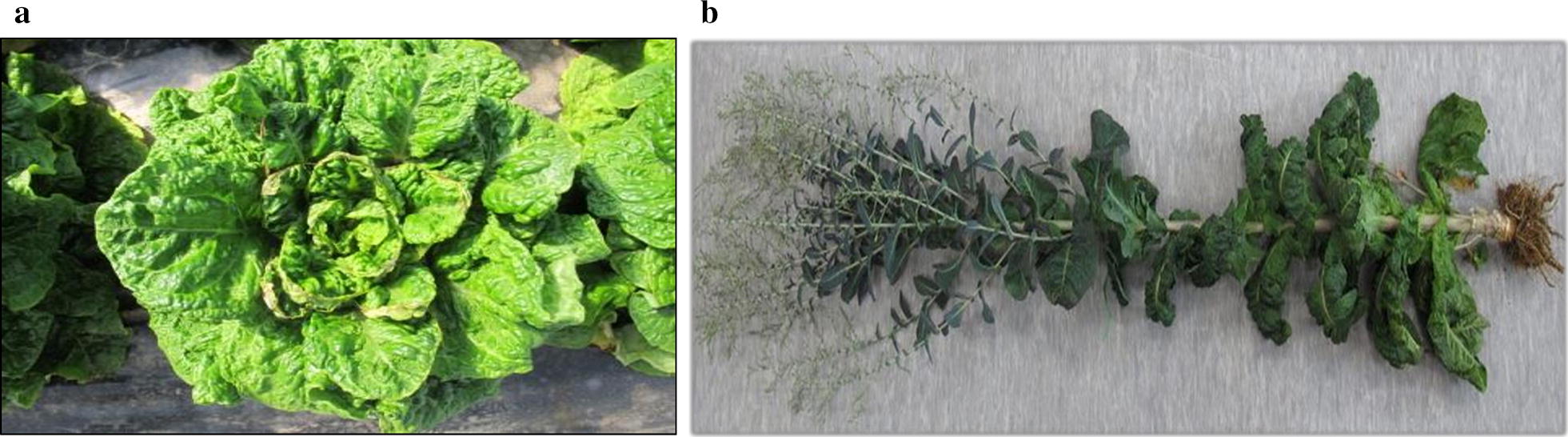
Representative photos of lettuce at mature stage (**a**) and bolting stage (**b**)

Lettuce were grown at the research farm of the National Agrobiodiversity Center (NAC), Rural Development Administration (RDA), Jeonju (35°49′18″N, 127°08′56″E), Republic of Korea. Seeds of 22 lettuce cultivars (18 cultivars of RDA breeding lines and four commercial cultivars originated from Korea) obtained from RDA gene bank were sown in plug trays, and seedlings were grown in a greenhouse. Four weeks old seedlings were transplanted to the field of a plastic house with planting density of 20 × 20 cm. RDA’s recommended cultural management practices for lettuce were followed in the field. Each cultivar consisted of 24 plants. Plant growth was maintained using nutrient solution throughout the growing season. Mature lettuce leaf sample, where the head/leaf reaches marketable size, were harvested 75 days after sowing while the bolting stage samples were harvested 120 days after sowing when the elongated stem has produced inflorescences and flowers. These samples were immediately transported to the laboratory. Figure 1 shows a representative photo of lettuce at the mature and bolting stage. The qualitative characters were recorded based on the plant observation on the field and in the laboratory. The experimental design was completely randomized and was conducted in three biological replicates. Lettuce leaf samples were placed in vinyl freezer bags and held at − 80 °C. The frozen samples were subsequently lyophilized for 48 h using vacuum freeze-drier (Ilshibiobase, Rijssen, Netherlands). Freeze-dried samples were ground to a fine powder using a mortar and pestle, and held at − 80 °C for the subsequent experiment.

### Extraction and analysis of phenolic acids and flavonoids

The extraction procedure was adopted from Llorach et al. [5] with some modifications. Briefly, 0.25 g of lyophilized powdered sample was extracted with 10 mL of methanol/water/formic acid (25:22:3, v:v:v) mixture using a thermostatic shaking water bath (agitation rate 140 rpm) at a temperature of 40 °C for 3 h. Then, the extract was centrifuged at 5000×*g* for 15 min at 4 °C and the supernatant was recovered. The residue was further extracted following the same step described above. The obtained supernatants were combined, filtered through a 0.45-μm polytetrafluoroethylene (PTFE) filter (Millipore Ltd., Bedford, MA, USA), dissolved in an appropriate concentration, and used for subsequent analysis (LC–MS/MS analysis and ABTS assay).

### LC–MS/MS analysis

Analysis of phenolic acids and flavonoids were performed using an ultra-performance liquid chromatography (UPLC) system (Waters, Milford, USA) equipped with autosampler and photodiode array (PDA) detector. The mobile phase compositions were water with 0.1% formic acid (A) and acetonitrile with 0.1% formic acid (B) with a solvent flow rate of 0.4 mL/min. Sample injection volume was 2 µL. A gradient elution was followed from 8 to 15% B for the first 5 min, from 15 to 18% B for the next 5 min, and remaining gradient from 18 to 90% B for the last 5 min. A post run analysis was held for 5 min. The UV chromatograms were recorded at 330 nm. The metabolites eluted from the column were detected by a high-resolution tandem mass spectrometer SYNAPT G2 Si HDMS QTOF (Waters) in negative ion mode. The capillary voltage and the cone voltage were set at 1 kV and 40 V, respectively. Centroid MS^E^ mode was used to collect the mass spectrometry data. The primary scan ranged from 50 to 1200 Da and the scanning time was 0.2 s. All the parent ions were fragmented using 20–40 eV. The information on all fragments was collected and the time was 0.2 s. For accurate mass acquisition, leucine-enkephalin ([M-H]^−^ = 554.2615) at a flow rate of 10 μL/min was used as a lock mass by a lock spray interface. Data acquisition and analysis were controlled by Waters UNIFI V1.71 software. The scan range in MS and MS/MS modes was from 50 to 1200 m/z.

### Extraction, hydrolysis, and analysis of anthocyanidins

Anthocyanidins extraction and hydrolysis were conducted based on the methodology used in a previous report with some modification [1]. Briefly, 10 mg of finely powdered lyophilized lettuce was mixed with 1.5 mL of an aqueous methanol solution containing 2 N HCL (50 mL of methanol + 33 mL of water + 17 mL of 37% HCL). The mixture was sonicated for 15 min in a sonication water bath (300 w, 60 Hz) followed by centrifugation (10 min, 4000 rpm). The supernatant was filtered through a 0.45-µm PTFE filter (Millipore Ltd., Bedford, MA, USA) and transferred to a vial with a Teflon-lined screw cap. The vial was placed in a preheated thermostatic water bath and hydrolyzed at 100 °C for 60 min. The standard of cyanidin 3-*O*-glucoside chloride was hydrolyzed following a similar procedure described above to obtain the cyanidin. Hydrolyzed samples were cooled to room temperature, made up to 5 mL, and immediately analyzed using UPLC.

Agilent 1290 infinity II UPLC (Agilent Technologies, Santa Clara, CA, USA) system equipped with an autosampler and PDA detector was used for analysis of cyanidin while Agilent eclipse plus C18 (1.8 µm, 2.1 × 50 mm) column was used for separation. The column thermostat was maintained at 25 °C. The solvent system consisted of 0.1% formic acid in water (mobile phase A) and 0.1% formic acid in acetonitrile (mobile phase B). A gradient elution was followed from 8 to 15% B for the first 5 min, from 15 to 18% B for the next 5 min, and followed by a 2 min isocratic elution at 18% of B. A post run analysis was kept for 5 min. The sample injection volume and mobile phase flow rate was kept at 3 µL and 0.4 mL/min, respectively. The signal acquisition wavelength was set at 520 nm. The quantification of cyanidin was done using a calibration equation (Y = 12854X − 12.13, R^2^ = 0.9996; where Y stands for peak area and X for concentration) which was constructed using a standard of hydrolyzed cyanidin 3-*O*-glucoside chloride (cyanidin).

### Extraction, separation, and analysis of sesquiterpene lactones (SLs)

Samples were extracted based on the method described in a previous report [20] with some modifications. In brief, powdered lyophilized lettuce (0.25 g) was mixed to 100 mL methanol, refluxed at 65 °C for $1\frac{1}{3}$ h, and filtered through a Whatman #2 filter paper. The solvent was evaporated under reduced pressure using High Capacity Centrifugal Evaporator (Genevac, HT-4X, 5 mm Hg, 30–35 °C). The crude extract was then re-dissolved with water to a final volume of 20 mL and partitioned two times with 20 mL chloroform. The chloroform phase was dried over anhydrous magnesium sulfate and evaporated at reduced temperature and pressure using High Capacity Centrifugal Evaporator (Genevac, HT-4X, 5 mm Hg, 30–35 °C). The residue was re-dissolved in 0.4 mL of methanol/chloroform (1/2, v/v) AND the SLs separated using high-performance liquid chromatography (HPLC).

The analysis of SLs was performed using an Agilent 1260 infinity HPLC system (Agilent Technologies, Santa Clara, CA, USA) equipped with a degasser, autosampler, and PDA detector (SPD-M10A). A Phenomenex Luna C18 (5 µm, 250 × 4.6 mm) column, maintained at 30 °C, was used for separation of the SLs. The solvent system was comprised of water (mobile phase A) and acetonitrile (mobile phase B). The elution program was as follows: 0–3 min, 10% B; 5–15 min, 35% B; 15–25 min, 35–100% B, and 25–30 min, 100% B. The SLs were monitored at a wavelength of 256 nm. The flow rate and injection volume were kept at 0.8 mL/min and 20 µL, respectively. Quantification was done using calibration equations (lactucin, Y = 3938.6X − 4.7184 R^2^ = 1; lactucopicirin, Y = 2606.8X + 1.2828, R^2^ = 1; where Y stands for peak area and X for concentration) derived from the calibration curves of the corresponding standards.

### ABTS assay

2,2′-Azinobis-(3-ethylbenzothiazoline-6-sulfonic acid) (ABTS) radical scavenging activity was estimated using an improved ABTS decolorization assay described by Re et al. [21] with little modifications. In brief, an ABTS radical cation was generated by reacting 7 mM ABTS stock solution which was prepared using water with 2.45 mM potassium persulphate (final concentration) followed by an overnight incubation of the mixture in the dark at room temperature. The ABTS radical cation solution was further diluted with water to obtain an absorbance of 0.7 ± 0.02 at 734 nm. A test solution was prepared by mixing diluted ABTS radical cation solution (190 µL) with 10 µL of sample solution or standard. After 6 min, absorbance at 734 nm was determined using an Eon Microplate Spectrophotometer (Bio-Tek, Winooski, VT, USA). The potential of lettuce extract to scavenge ABTS radical was calculated using the following equation:

$${\text{ABTS scavenging activity}} = 1{\,-\,}({\text{A}}_{\text{sample}} {\,-\,}{\text{A}}_{\text{sample blank}} )/({\text{A}}_{\text{control}} {\,-\,}{\text{A}}_{\text{control blank}} )$$

A_sample_ is absorbance of test solution; A_sample blank_ is the absorbance of solvents used in the test solution; A_control_ is the absorbance of ABTS radical cation solution; and water is used as A_control blank_. The radical scavenging activity was reported as mg TROLOX equivalent per gram (mg TE/g) DW obtained by comparing the results with a TROLOX calibration curve. The calibration equation used for quantification was Y = 6.4879X + 0.0024 (where Y stands for absorbance and X for concentration; R^2^ = 0.9983).

### Statistical analysis

Results were expressed as mean ± standard deviation (SD) of biological triplicates (each replicate tested three times) based on the dry weight (DW) of the lyophilized sample. The data were analyzed using a one-way ANOVA with SPSS 17.0 software followed by Duncan’s multiple range test using SPSS V. 17.0 statistical program (SPSS Inc., Chicago, USA). Significant level was set at p < 0.05. The correlation between ABTS radical scavenging activity and cyanidin content of lettuce was determined by two-tailed Pearson correlation analysis (*p* < 0.01) with the same program. The multivariate analysis was performed using PAST (Palaeontological statistics, version 3.06) [22] to create a principal component analysis (PCA) score and loading plots.

## Results and discussion

Differently pigmented (green, green/red, and red) lettuce plants belonging to four different growth types including, crisphead, butterhead, cos (romaine), and leafy, harvested at the mature and bolting stages, were evaluated for their metabolites compositions and antioxidant potential. The extraction and analysis of phenolics and flavonoids, cyanidin, and sesquiterpene lactones were conducted using separate procedures to ensure a maximum throughput of each group of compounds. Samples were lyophilized before extraction because freeze drying allows highest recovery of polyphenols in previous studies [23].

### Identification of phenolic acids and flavonoids in lettuce cultivars

**Fig. 2:**
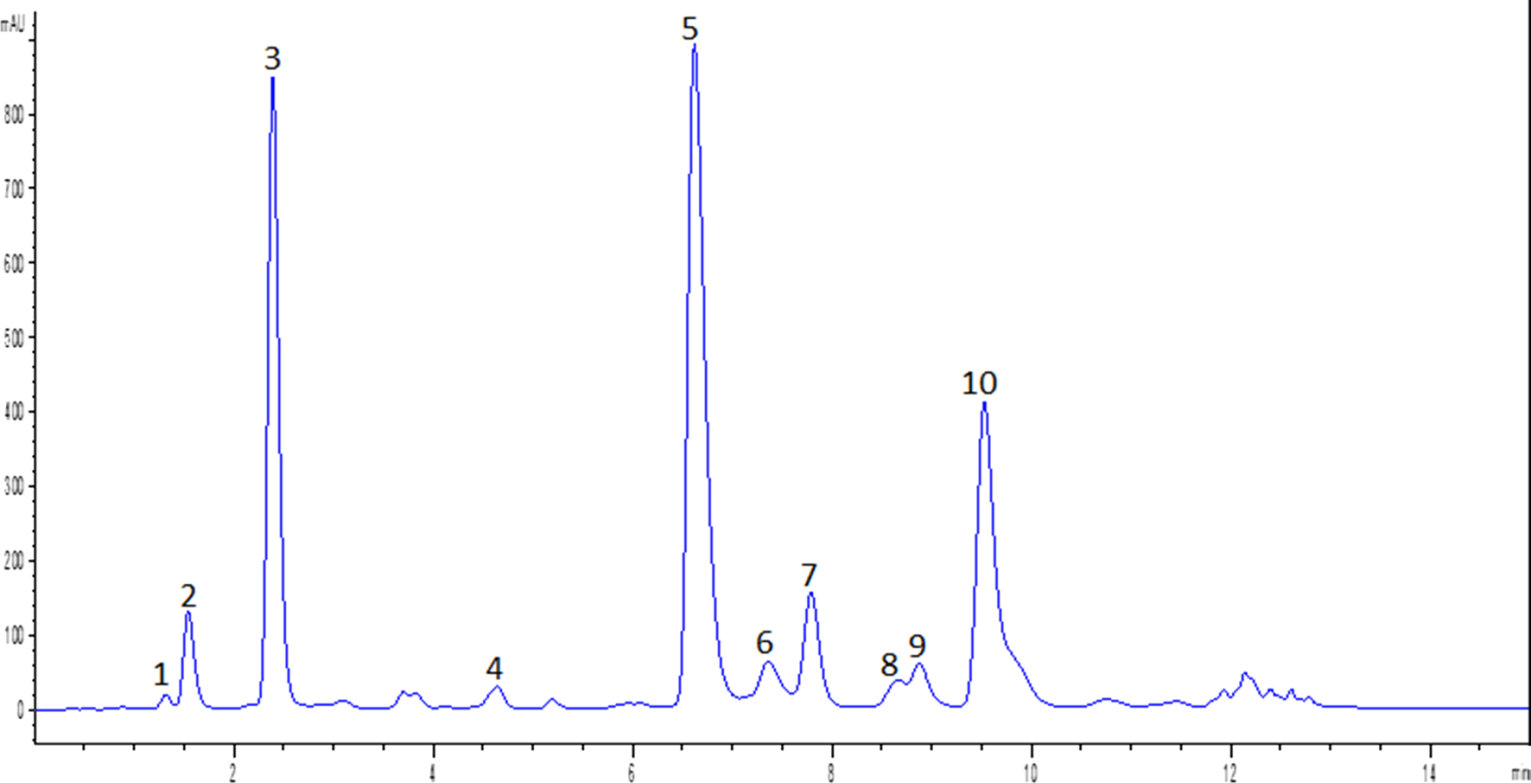
Representative chromatogram of identified compounds in lettuce cultivars. The peak numbers correspond to the identified compounds as presented in Table 1

The identification and characterization of phenolic acids and flavonoids for which standards were available were performed by comparison to their UV/Vis spectra, retention times, and the MS and MS/MS fragmentation patterns of the ions recorded in negative ion mode. The other compounds were elucidated based on the available data on literature, the UV/Vis spectrum when it was available to assign a phenolic class and the deprotonated dimer ion [2M-H]^–^. The identities, retention times, UV absorption maxima’s, and observed molecular and fragment ions of the identified compounds from lettuce cultivars by UPLC-PDA-Q-TOF-HDMS are presented in Table 1. Reversed-phase liquid chromatography using gradient acidic water and acetonitrile mobile phase conditions have been employed for separation of phenolic compounds in lettuce samples in earlier reports [8]. The chromatographic conditions allowed a baseline separation of the peaks of the phenolic compounds in 15 min. A representative chromatogram of the identified compounds is presented in Fig. 2. Three peaks (1, 2, and 3) presented almost similar UV spectra and gave the same [M-H]^–^ ion at *m/z* 353, fragment ion at *m/z* 191 ([quinic acid–H]^–^), and deprotonated dimer ion ([2M-H]^–^) at *m/z* 707. Those peaks were identified as monocaffeoylquinic acids. MS/MS spectra did not show fragment ion at m/z 173 ([quinic acid–H–H_2_O]^–^), characteristically formed in the negative ion mode when the cinnamoyl group is bonded to the quinic moiety at position 4 of caffeoylquinic acid structure [24], confirming the absence of 4-*O*-caffeoylquinic acid. Peak **3** was unambiguously identified as 5-*O*-caffeoylquinic acid (5-CQA) by comparison to its UV/Vis spectra, retention time, and the patterns of MS and MS/MS fragmentations of the standard. Thus, peaks **1** and **2** were respectively identified as 1-*O*-caffeoylquinic acid (1-CQA) and 3-*O*-caffeoylquinic acid (3-CQA) based on their MS parent ion, deprotonated dimer ion, MS/MS product ions, and by comparing with the elution order of caffeoylquinic acids in earlier reports [25, 26]. 1-CQA, 3-CQA, and 5-CQA have been already reported in lettuce [24]. Peak **4** presented MS parent ion at *m/z* 367 and fragment ions at m/z 191 [quinic acid]^–^, 173 [quinic acid–H_2_O–H^+^]^–^, 149 [ferulic acid ion–COO]^–^, and 134[ferulic acid ion–COO–CH_3_]^–^. The MS/MS base peak at m/z 191 indicate a C-5 substituent [27]. Hence, this peak was assigned as 5-feruloylquinic acid (5-FQA) by comparison to the fragmentation behavior and UV spectra of previous reports [28–30]. The presence of 5-FQA has also been previously reported in green lettuce [31]. Peak **5**, which presented MS parent ion at m/z 473, was identified as dicaffeoyltartaric acid (DCTA) on the basis of its concordance with retention time, UV/Vis spectra, MS, and MS^2^ fragmentation pattern of the authentic standard. Similar to previous studies [5, 17, 24], DCTA was found to be the most abundant phenolic acid in lettuce. Peaks **6**, **9**, and **10** were identified as kaempferol derivatives namely kaempferol glucuronide (KGR), kaempferol malonylglucoside (KMG), and methylkaempferol glucuronide (MKGR), respectively, on the basis of UV/Vis and mass spectral data of the previous reports [5, 11, 24, 32]. The [M-H]^−^ at *m/z* 461 and [Y_o_]^−^ at m/z 285 of peak **6** were characteristic properties of KGR. Though luteolin and kaempferol aglycone are isobaric, their derivatives can be distinguished based on MS^n^ data [24]. Peak **6** yielded the base peak at *m/z* 285 ([Yo]^−^) at the high energy function indicating it is kaempferol derivative. In the low energy function, peak **9** presented [M-H]^−^ at *m/z* 533 and a base peak at *m/z* 489 due to loss of CO_2_. The high energy function yielded a base peak at *m/z* 285 ([Yo]^−^), suggesting that this compound is a kaempferol derivative. Based on the UV/Vis and mass spectral information, peak **9** was identified as KMG. Glucosides, glucuronides, and malonylglucosides of kaempferol were already detected in lettuce [5, 11, 24, 32]. Peak **10** presented [M-H]^–^ at m/z 475 as a base peak and a dimer ion at *m/z* 951. The MS/MS yielded fragment ions at m/z 299, 285, and 284 which could be due to the loss of glucuronide moiety from the parent ion, kaempferol aglycone, and loss of the methyl group from the ion at m/z 299, respectively. Hence, this peak was tentatively identified as methylkaempferol glucuronide. The methyl conjugate of kaempferol glucuronide could result from methylation with methanol during the methanol/water/formic acid mixture extraction process as observed in the previous report [33]. Peak **7** was unambiguously identified as quercetin malonylglucoside (QMG) by comparison to the retention time, UV/vis and mass spectra of the authentic standard. It presented [M-H]^−^ at *m/z* 549, [Y_o_-H]^−^ at 300, and [M-H–CO_2_]^−^ (base peak) at m/z 505, which are the characteristic properties of QMG. As it was previously pointed out by Abu-reidah et al. [34], the loss of CO_2_ was a common feature of compounds presenting the malonyl group due to in-source fragmentation. This caused the [M-H]^−^ to appear in lower abundance in the mass spectra than the product ion [M-H−CO_2_]^−^ as observed for peaks **7** and **9**. The identification of QMG which has been previously detected in lettuce [5, 7, 34, 35], was further confirmed by the presence of [2M-H]^−^ ion at *m/z* 1099. Peak **8** which presented [M-H]^−^ at *m/z* 515, was identified as dicaffeoylquinic acid (DCQA). The first MS/MS fragment of DCQA was due to the loss of one of the caffeoyl moieties leading to the precursor ion of caffeoyl quinic acid (at *m/z* 353). The subsequent fragmentations presented similar ions as described above for peaks **1**, **2** and **3**. The identification of DCQA, also reported in lettuce [5, 7, 24, 34], was further supported by the presence of dimer ion at *m/z* 1031.

**Table 1:** Retention times, ultraviolet–visible absorption maxima, and mass spectral data of phenolic acids and flavonoids in leaves of lettuce cultivars

Peak no	t_R_ (min)	UV λ_max_ (nm)	Neutral mass (Da)	Observed m/z [M-H]	Dimer and fragment ions in ESI/MS/MS	Mass error, ppm	Identification
1	1.32	300sh, 326	354.0951	353.08698	707.18302 [2M-H]^−^, 191.05512 [Quin-H]^−^, 85.02891	− 2.3	1-*O*-Caffeoylquinic acid (1-CQA)
2	1.54	300sh, 324	354.0951	353.08674	707.18124 [2M-H]^−^, 191.05503 [Quin-H]^−^, 179.03365	− 3.0	3-*O*-Caffeoylquinic acid (3-CQA)
3	2.39	300sh, 324	354.0951	353.08697	707.18302[2M-H]^−^, 191.05512[Quin-H]^−^, 85.02891	− 2.4	5-*O*-Caffeoylquinic acid (5-CQA)
4	4.64	328	368.1107	367.1025	191.05493, 134.03524	− 2.6	5-Feruloylquinic acid (5-FQA)
5	6.62	301sh, 324	474.0798	473.0719	947.15066 [2M-H]-, 311.03986 [Caftar-H]^−^, 293.02929 [Caftar-H-H_2_O]^−^, 179.03366 [Caffeic–H]^−^, 149.00859 [Tartaric–H]^−^ 133.02836, 112.98729	− 1.4	Dicaffeoyltartaric acid (DCTA)
6	7.36	328	462.0798	461.07175	285.03951, 133.02837	− 1.7	Kaempferol glucuronide (KGR)
7	7.79	252, 360	550.09587	549.08832	1099.18568 [2M-H]^−^, 505.09879 [M-H-CO_2_], 300.02672 [Y_o_-H], 271.02369, 255.02863 [Y_o_-CHO-OH]^−^	− 0.5	Quercetin malonylglucoside (QMG)
8	8.66	330	516.1268	515.1190	1031.24625 [2M-H], 353.08678[Cafquin-H], 191.05486 [Quin-H]-, 179.03376 [Caffeic-H]-, 135.4416 [Caffeic-H–CO_2_]	− 1.0	Dicaffeoylquinic acid (DCQA)
9	8.88	348	534.1009	533.09327	489.10292, 285.03897 [luteolin-H]-284.03133, 133.02831	− 0.7	Kaempferol malonylglucoside (KMG)
10	9.53	328	476.0955	475.08749	951.18882 [2M-H], 299.05492, 285.03121, 284.03121	− 1.5	Methylkaempferol glucuronide (MKGR)

### Quantitative variations of metabolites and antioxidant activity among 22 cultivars at mature and bolting stages

To investigate the quantitative variability of metabolites in lettuce cultivars, representative leaf samples were prepared for each cultivar at the mature and bolting stages. Table 2 shows the phenolic acid and flavonoid contents of 22 lettuce cultivars expressed as mg/100 g of dry weight (DW). Calibration curves were created using authentic standards (5-CQA, DCTA, KG, and QMG) for calculations of results. Among the ten compounds identified using UPLC-PDA-Q-TOF-HDMS, eight of them showing prominent peaks were quantified. For quantification of compounds with no standards available, the most similar compounds were used taking into account the nature of the compounds and their molecular weight. Hence, 3-CQA and 5-FQA were quantified using a calibration curve of 5-CQA whereas kaempferol derivatives were quantified using KG. To investigate the relationship between leaf age and phenolic compositions of lettuce, the individual phenolic acids and flavonoids in lettuce at mature (marketable stage) and bolting stages were quantified. The main phenolic acid components of lettuce in this study were quinic and tartaric acid derivatives where 5-CQA and DCTA contributed 20 to 74% and 15 to 56% of the total content of the sum of phenolic acids determined, respectively. The maximum and minimum content of phenolic acids (sum of individual phenolic acids quantified) in lettuce at the mature stage was found in a green pigmented cultivars “Chunpungjeokchukmyeon” (54.6 mg/100 g DW) and “Adam” (18.3 mg/100 g DW), respectively. At the bolting stage, red pigmented commercial cultivar, “Tomalin”, had the highest amount of phenolic acid (54.6 mg/100 g DW) while green pigmented “Pungseong” cultivar contained the least amount (15.5 mg/100 g DW). Kaempferol derivatives were the major flavonoids detected in lettuce samples. There is also a significant content of QMG measured in lettuce especially at the bolting stage. The sum of flavonoids quantified in this study ranged from 9.2 to 25.9 mg/100 g DW and 14.9 to 83.0 mg/100 g DW in mature and bolting stage lettuce samples, respectively. We have noticed that 3-CQA, KMG, and MKGR were higher (up to 300%) at the bolting than the mature stage for most of the samples. Lettuce leaves at the bolting stage exhibited significantly higher (up to 12.5-fold) QMG than at the mature stage. Unlike other cultivars, “Cheonsang”, a green colored romaine lettuce, showed two times less QMG at the mature stage compared to the bolting stage. However, at least 70% of the samples showed up to 4.5-, 3.2-, and 3.0-times higher content of 5-CQA, 5-FQA, and KGR at the mature stage compared to bolting stage leaves, respectively. In several samples, 1-CQA and DCQA were below the level of the limit of quantification, thus the quantitative information is not included in this report. In this study, the most commonly found phenolic acids are represented by chlorogenic acids and chicoric acids while the flavonoids constitute glucuronide and glucoside conjugates of quercetin and kaempferol which was in agreement with previous reports [6, 17, 24].

**Table 2:** Types of lettuce cultivars and contents of individual phenolic acids and flavonoids at mature and bolting stages

S/No	Cultivar	Cultivar type	Leaf color	3-CQA		5-CQA		5-FQA	
				Mature stage	Bolting stage	Mature stage	Bolting stage	Mature stage	Bolting stage
1	Adam	Crisphead	Green	3.99 ± 0.05f	2.51 ± 0.04b	0.87 ± 0.04a	9.85 ± 0.06abc	9.85 ± 0.06abc	1.79 ± 0.04cde
2	Pungseong	Crisphead	Green	4.4 ± 0.7fg	1.72 ± 0.02a	2.6 ± 0.3def	10.6 ± 1.6bcde	10.6 ± 1.6bcde	1.62 ± 0.02bcd
3	Jeokhagye	Leaf	Green/red	3.22 ± 0.04e	3.9 ± 0.2h	3.17 ± 0.04gh	17.6 ± 0.2ij	17.6 ± 0.2ij	4.4 ± 0.3j
4	Jeoksagye	Leaf	Green/red	4.8 ± 0.1ghi	4.4 ± 0.1i	1.84 ± 0.06bc	15.9 ± 0.5hi	15.9 ± 0.5hi	4.5 ± 0.3j
5	Cheonsang	Romaine	Green	5.1 ± 0.2i	5.6 ± 0.4k	0.70 ± 0.03a	9.4 ± 0.3abc	9.4 ± 0.3abc	0.59 ± 0.04a
6	Mansang	Romaine	Green	4.57 ± 0.02gh	3.37 ± 0.06ef	0.84 ± 0.00a	13.61 ± 0.04g	13.61 ± 0.04g	1.03 ± 0.03ab
7	Hacheong	Leaf	Green	4.80 ± 0.07ghi	6.2 ± 0.3l	1.60 ± 0.02b	18.9 ± 0.2j	18.9 ± 0.2j	1.2 ± 0.1bc
8	Jeokdan	Leaf	Green/red	5.00 ± 0.04hi	5.57 ± 0.03k	2.30 ± 0.02cde	16.6 ± 0.1i	16.6 ± 0.1i	2.87 ± 0.03h
9	Jangsu	Leaf	Green/red	1.90 ± 0.05b	3.6 ± 0.1fg	3.68 ± 0.09ij	10.4 ± 0.3bcd	10.4 ± 0.3bcd	2.8 ± 0.1fgh
10	Gopungjeokchukmyeon	Leaf	Green/red	2.04 ± 0.02bc	3.05 ± 0.06cd	3.49 ± 0.05hi	12.8 ± 0.2efg	12.8 ± 0.2efg	2.73 ± 0.06fgh
11	Mihong	Leaf	Red	2.55 ± 0.04cd	3.41 ± 0.08ef	2.2 ± 0.2cd	14.4 ± 1.7gh	14.4 ± 1.7gh	2.18 ± 0.06def
12	Chunpungjeokchukmyeon	Leaf	Green/red	1.34 ± 0.07a	2.51 ± 0.06b	5.2 ± 0.6l	8.3 ± 0.8ab	8.3 ± 0.8ab	2.65 ± 0.06fgh
13	Gohong	Leaf	Red	1.88 ± 0.01b	5.03 ± 0.07j	3.11 ± 0.06fgh	11.1 ± 0.2cdef	11.1 ± 0.2cdef	2.32 ± 0.03efgh
14	Miseonjeokchukmyeon	Leaf	Red	2.21 ± 0.03bcd	4.56 ± 0.05i	2.76 ± 0.06efg	13.3 ± 0.2fg	13.3 ± 0.2fg	3.98 ± 0.01ij
15	Sunredbutter	Butterhead	Green/red	3.22 ± 0.02e	5.55 ± 0.09k	1.38 ± 0.01b	14.0 ± 0.1gh	14.0 ± 0.1gh	1.45 ± 0.02bc
16	Hyeseonmanchudae	Leaf	Green/red	2.50 ± 0.05cd	5.04 ± 0.04j	4.04 ± 0.07j	12.6 ± 0.2defg	12.6 ± 0.2defg	2.81 ± 0.02gh
17	Sambokhacheong	Leaf	Green	2.30 ± 0.02bcd	2.28 ± 0.00b	2.51 ± 0.01de	9.4 ± 0.3abc	9.4 ± 0.3abc	1.75 ± 0.00cde
18	Chunhachujeokchima	Leaf	Red	1.91 ± 0.02b	2.96 ± 0.04c	2.46 ± 0.06de	9.4 ± 0.3abc	9.4 ± 0.3abc	1.45 ± 0.04bc
19	Yelpungjeokchima	Leaf	Green/red	2.7 ± 0.2d	3.9 ± 0.2gh	2.7 ± 0.2efg	11.0 ± 0.6cde	11.0 ± 0.6cde	3.8 ± 0.2i
20	Superseonpung	Leaf	Red	2.5 ± 0.5cd	3.3 ± 0.3de	4.6 ± 0.1k	12.8 ± 0.7efg	12.8 ± 0.7efg	5.2 ± 0.8k
21	Tomalin	Leaf	Red	1.3 ± 0.3a	2.4 ± 0.4b	4.0 ± 0.5ij	7.7 ± 0.8a	7.7 ± 0.8a	2.2 ± 0.4efg
22	Cheongchima	Leaf	Green	2.7 ± 0.4d	4.6 ± 0.6i	1.9 ± 0.2bc	7.8 ± 1.1a	7.8 ± 1.1a	1.4 ± 0.2bc

S/No	DCTA		KGR		QMG		KMG		MKGR	
	Mature stage	Bolting stage	Mature stage	Bolting stage	Mature stage	Bolting stage	Mature stage	Bolting stage	Mature stage	Bolting stage
1	9.85 ± 0.06abc	5.9 ± 0.2a	5.4 ± 0.8ab	3.0 ± 0.2a	1.6 ± 0.2a	16.1 ± 0.4d	0.56 ± 0.08ab	1.73 ± 0.04f	1.7 ± 0.9a	5.3 ± 0.9ef
2	10.6 ± 1.6bcde	5.51 ± 0.08a	6.5 ± 1.0bc	3.2 ± 0.1a	3.6 ± 0.6b	20.7 ± 0.3e	0.68 ± 0.08b	1.45 ± 0.01de	2.6 ± 2.08bc	4.0 ± 0.2cdef
3	17.6 ± 0.2ij	13.5 ± 0.7gh	12.1 ± 0.2f	8.1 ± 0.4gh	ND	28.2 ± 1.8gh	1.30 ± 0.01ef	1.88 ± 0.08f	7.5 ± 0.4m	11.5 ± 3.2i
4	15.9 ± 0.5hi	10.2 ± 0.3e	11.8 ± 0.4f	5.4 ± 0.1cd	ND	24.5 ± 0.8f	1.16 ± 0.03def	2.4 ± 0.1g	3.7 ± 0.6efg	10.4 ± 1.9hi
5	9.4 ± 0.3abc	13.9 ± 0.8ghi	4.3 ± 0.2a	8.7 ± 0.5h	6.2 ± 0.3d	3.1 ± 0.2a	1.63 ± 0.06g	0.65 ± 0.03a	1.4 ± 0.4a	2.8 ± 1.0abcd
6	13.61 ± 0.04g	8.2 ± 0.1c	8.58 ± 0.02de	5.30 ± 0.08cd	4.65 ± 0.02bc	18.4 ± 2.0de	1.15 ± 0.01def	1.31 ± 0.02bcd	2.4 ± 0.07b	2.1 ± 0.2abc
7	18.9 ± 0.2j	14.4 ± 0.6hij	12.8 ± 0.3f	7.6 ± 0.4fgh	ND	17.5 ± 1.6d	0.94 ± 0.01cd	1.21 ± 0.05b	5.8 ± 0.4l	5.9 ± 1.3f
8	16.6 ± 0.1i	11.8 ± 0.2f	12.3 ± 0.9f	7.5 ± 0.1fg	ND	18.2 ± 0.2d	1.24 ± 0.02ef	2.35 ± 0.02g	3.00 ± 0.05bcde	8.0 ± 0.5g
9	10.4 ± 0.3bcd	15.2 ± 0.6jk	6.6 ± 0.2bc	8.7 ± 0.4h	5.6 ± 0.2cd	35.2 ± 1.1i	1.20 ± 0.03ef	1.45 ± 0.05de	4.9 ± 0.7jk	9.2 ± 1.7gh
10	12.8 ± 0.2efg	11.6 ± 0.2f	8.3 ± 0.1de	5.9 ± 0.1cde	9.8 ± 0.1fgh	42.5 ± 0.7k	1.70 ± 0.02g	1.82 ± 0.02f	5.5 ± 0.4kl	4.1 ± 1.2cdef
11	14.4 ± 1.7gh	11.3 ± 0.5f	8.9 ± 0.9de	5.8 ± 0.3cd	5.9 ± 0.9cd	46.5 ± 1.4l	1.4 ± 0.1f	1.87 ± 0.02f	4.9 ± 1.2jk	4.8 ± 0.8def
12	8.3 ± 0.8ab	11.8 ± 0.3f	5.8 ± 0.7bc	6.0 ± 0.2cde	9.6 ± 1.4fg	28.7 ± 0.7h	1.67 ± 0.13g	1.87 ± 0.04f	4.5 ± 2.5hij	3.4 ± 0.4abcde
13	11.1 ± 0.2cdef	18.6 ± 0.2l	7.4 ± 0.2cde	8.3 ± 0.1gh	10.2 ± 0.3gh	26.0 ± 0.4fg	1.69 ± 0.01g	2.24 ± 0.02g	4.8 ± 0.4ijk	4.4 ± 0.2def
14	13.3 ± 0.2fg	13.1 ± 0.2g	9.0 ± 0.1e	8.5 ± 0.1gh	7.9 ± 0.1e	40.6 ± 0.4k	1.26 ± 0.01ef	1.38 ± 0.02cde	4.7 ± 0.4ij	10.7 ± 0.3hi
15	14.0 ± 0.1gh	13.6 ± 0.1gh	8.17 ± 0.09de	6.42 ± 0.07de	8.52 ± 0.08ef	12.4 ± 0.2c	0.91 ± 0.01c	0.65 ± 0.01a	2.90 ± 0.09bcd	1.7 ± 0.1ab
16	12.6 ± 0.2defg	15.4 ± 0.1k	8.6 ± 0.2de	8.15 ± 0.08gh	5.3 ± 0.1cd	38.1 ± 0.2j	1.14 ± 0.02def	1.28 ± 0.02bc	4.1 ± 0.4ghi	10.1 ± 0.5hi
17	9.4 ± 0.3abc	7.79 ± 0.02bc	6.5 ± 0.2bc	4.85 ± 0.02bc	2.38 ± 0.03a	29.8 ± 0.2h	0.65 ± 0.01b	0.56 ± 0.00a	3.7 ± 0.2efg	3.6 ± 2.0abcde
18	9.4 ± 0.3abc	11.5 ± 0.2f	6.5 ± 0.3bc	5.62 ± 0.07cd	4.5 ± 0.2bc	16.3 ± 0.3d	1.07 ± 0.01cde	1.48 ± 0.01e	3.2 ± 0.3cdef	3.7 ± 0.4bcde
19	11 ± 0.6cde	14.7 ± 0.8ijk	7.3 ± 0.5cd	7.0 ± 1.3ef	5.0 ± 0.3cd	45.0 ± 3.0l	0.89 ± 0.03c	1.37 ± 0.07cde	4.5 ± 1.4hij	13.9 ± 5.7k
20	12.8 ± 0.7efg	7.0 ± 1.0b	9.0 ± 0.2e	3.00 ± 0.08a	9.7 ± 0.2fgh	28.9 ± 1.6h	1.6 ± 0.2g	2.720.3h	5.5 ± 1.2kl	11.2 ± 6.6hi
21	7.7 ± 0.8a	10.9 ± 1.5ef	5.9 ± 0.5bc	3.9 ± 0.3ab	11.0 ± 1.2h	67.2 ± 4.1m	1.82 ± 0.04g	2.3 ± 0.1g	3.9 ± 0.7fgh	9.6 ± 1.6ghi
22	7.8 ± 1.1a	9.2 ± 1.1d	5.3 ± 0.4ab	5.0 ± 0.6c	1.67 ± 0.01a	7.8 ± 1.2b	0.39 ± 0.06a	0.51 ± 0.05a	3.4 ± 0.9def	1.6 ± 1.1a

Results are expressed as mg/100 g DW

Values are Mean  ±  standard deviation of biological triplicates. Different letters between rows indicate statistically significant differences at *p*  < 0 0.05

*S/No* sample number, *3-CQA* 3-*O*-caffeoylquinic acid, *5-CQA* 5-*O*-caffeoylquinic acid, *5-FQA* feruloylquinic acid, *DCTA* dicaffeoyltartaric acid, *KGR* kaempferol glucuronide, *QMG* quercetin malonylglucoside, *DCQA* dicaffeoylquinic acid, *KMG* kaempferol malonylglucoside, *MKGR* methylkaempferol glucuronide, *ND* not detected

**Fig. 3:**
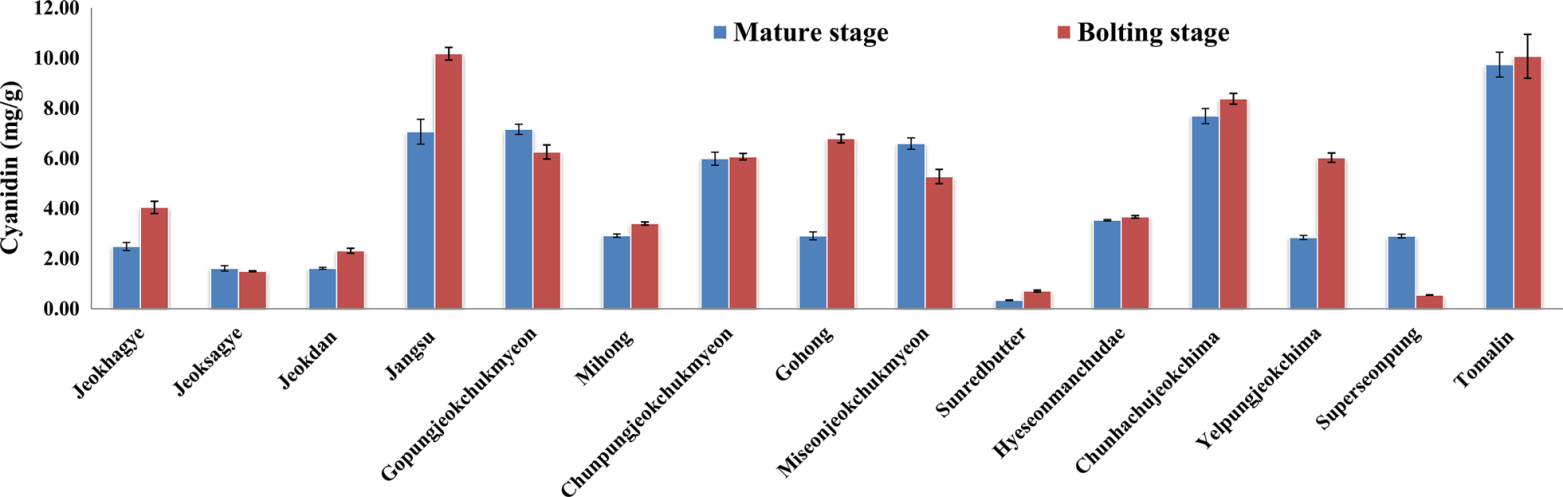
The content of cyanidin in red and green/red pigmented lettuce cultivars

Anthocyanins are responsible for the red pigments in lettuce [1, 4]. Anthocyanins exhibit specific UV/Vis absorption maxima at about 520 nm. The identification of anthocyanidins (an acid hydrolyzed form of anthocyanin) was conducted by comparing the retention time and UV/Vis spectra of acid hydrolyzed cyanidin 3-*O*-glucoside chloride standard. The anthocyanins were quantified after acid hydrolysis, which could convert these pigments into aglycone form. Cyanidin was the single major anthocyanidin detected in this study. The content of cyanidin in red and green/red pigmented lettuce cultivars is presented in Fig. 3. However, cyanidin was not detected in green pigmented lettuce cultivars. The cyanidin content was ranged from 0.3 (“Sunredbutter”) to 9.7 mg/g DW (“Tomalin”) in mature stage lettuce and 0.5 (“Superseonpung”) to 10.2 mg/g DW (“Jangsu”) in bolting stage lettuce. A statistically significant difference (*p* < 0.05) in cyanidin content was observed among red and green/red cultivars of lettuce. The cyanidin content in some of the cultivars of this study is quite higher than an earlier report by Kim et al. [1], which recorded 0.08 to 3.66 mg/g DW in baby-sized green/red and red lettuce leaves. Llorach et al. [5] found anthocyanin level of 0.259 and 0.456 mg/g (fresh weight basis) in “red oak leaf” and “lollo rosso” samples, respectively. In contrast, Pérez-López et al. [6], recorded quite higher cyanidin content (higher than 30 mg/g DW) in red pigmented lettuce cultivar. There was also a significant difference in cyanidin content between lettuce at the mature and bolting stages in some of the cultivars. An increase in cyanidin content was recorded at the bolting stage for some of lettuce cultivars (greater than 100% for “Gohong”, “Sunredbutter”, and “Yelpungjeokchima” and 43 to 63% for “Jangsu”, “Jeokdan”, and “Jeokhagye”) while a decrease by 81% was exhibited in “Supersongpung”. For the other cultivars, the increase/decrease in cyanidin content at the reproductive stage was not more than 20%. It has been previously reported that the cyanidin level of lettuce is influenced by the red color intensity, genotype, temperature, and growing conditions [4, 14, 36]. The amount of cyanidin accumulation is related to the degree of leaf redness, suggesting the visual assessment of the redness intensity could help as a measure of the relative quantity of cyanidin in lettuce. As reported earlier [12, 37], cyanindin 3-malonylglucoside is the principal anthocyanin in red pigmented lettuce. In addition to providing sensorial characteristics of food products [38], cyanidin 3-malonylglucoside in red lettuce has been shown to possess several health properties such as mitigating photoinhibitory and photooxidative damage [39].

SLs are C-15 terpenoids that naturally occur in the form of hydrocarbons, alcohols, ketones, aldehydes, acids or lactones [40]. Lactucin and lactucopicrin, the major SLs reported in lettuce, contribute significantly to the bitterness nature of lettuce cultivars [3, 20]. Two bitter sesquiterpene lactones (SLs), lactucin and lactucopicrin, were identified and quantified using HPLC by comparing their retention time and UV/Vis spectra to their corresponding authentic standards. Table 3 presents the mean SLs concentrations, expressed in microgram per gram of dry weight (µg/g DW), of 22 cultivars cultivated at mature and bolting stages. Analysis of variance (ANOVA) indicated that individual SLs, as well as total SLs concentration, showed significant inter-cultivar variations. The concentration of total SLs (sum of lactucin and lactucopicrin) ranged from 11.7 (“Superseonpung”) to 386.7 (“Sunredbutter”) µg/g DW and 213.0 (“Cheonsang”) to 4101.2 (“Superseonpung”) µg/g DW at the mature and bolting stage, respectively. Since all accessions were grown under similar agronomic conditions, the variations are likely to be genetically controlled. Analysis of SLs including, 8-deoxylactucin, jacquinelin, crepidiaside B and lactuside showed mixed result among 23 accessions of *Lactuca aculeata* Boiss. grown under standardized glasshouse conditions, suggesting that the genetic factor plays a major role [41]. Another study also showed that the concentration of lactucin (2.9 to 17.2 µg/g DW) and lactucopicrin (8.8 to 36.1 µg/g DW) varied significantly among cultivars due to leaf color and morphology [3]. The content of SLs was found to exhibit a significant variability based on variety in chicory and endive [42].

**Table 3:** Concentration of sesquiterpene lactones (SLs) in 22 cultivars of lettuce at mature and bolting stages

S/No	Cultivar	Lactucin (µg/g)		Lactucopicrin (µg/g)		Total SLs (µg/g)	
		Mature stage	Bolting stage	Mature stage	Bolting stage	Mature stage	Bolting stage
1	Adam	5.2 ± 0.4efgh	174.8 ± 14.7b	29.4 ± 1.7l	615.1 ± 30.2gh	34.6 ± 1.7l	789.9 ± 36.5ef
2	Pungseong	2.2 ± 0.1kl	181.2 ± 3.0b	29.3 ± 2.7l	1255.8 ± 12.3cd	31.5 ± 2.1l	1437.1 ± 11.3bc
3	Jeokhagye	5.3 ± 0.2efg	99.8 ± 6.8d	80.4 ± 1.8d	1156.9 ± 64.5de	85.8 ± 1.5e	1256.7 ± 57.7bcd
4	Jeoksagye	6.6 ± 0.4e	81.7 ± 3.3ef	64.7 ± 3.1fg	1396.7 ± 29.0bc	71.3 ± 2.6fgh	1478.4 ± 24.8bc
5	Cheonsang	13.7 ± 0.2c	43.8 ± 2.2g	52.2 ± 3.7hijk	169.2 ± 9.9i	65.9 ± 3.2hi	213.0 ± 9.8h
6	Mansang	14.9 ± 1.4c	80.4 ± 2.6ef	61.8 ± 3.8fg	207.8 ± 9.1i	76.7 ± 4.0efg	288.2 ± 9.5gh
7	Hacheong	6.1 ± 0.3ef	35.8 ± 1.8ghi	99.0 ± 6.5c	1176.4 ± 112.6de	105.1 ± 5.4d	1212.2 ± 93.2cd
8	Jeokdan	9.8 ± 0.9d	84.9 ± 6.4def	124.1 ± 3.3b	1227.9 ± 81.6cd	133.9 ± 3.4c	1312.8 ± 70.7bcd
9	Jangsu	3.2 ± 0.3ijkl	42.3 ± 1.4gh	45.9 ± 2.1k	593 ± 23.8gh	49.1 ± 1.8k	635.2 ± 20.6f
10	Gopungjeokchukmyeon	2.7 ± 0.2jkl	9.7 ± 0.6j	60.6 ± 2.8fghi	815.3 ± 18.6fg	63.4 ± 2.4hij	825.1 ± 15.7ef
11	Mihong	2.2 ± 0.1kl	11.1 ± 0.8j	62.5 ± 2.5fg	579.2 ± 31.4h	64.7 ± 2.1hi	590.3 ± 26.3f
12	Chunpungjeokchukmyeon	2.2 ± 0.2kl	18.0 ± 0.6ij	61.4 ± 4.4fgh	628.2 ± 55.9gh	63.6 ± 3.7hij	646.2 ± 46.0f
13	Gohong	3.6 ± 0.2hijk	17.8 ± 0.6ij	64.7 ± 6.9fg	662.6 ± 57.9gh	68.3 ± 5.8gh	680.4 ± 47.3f
14	Miseonjeokchukmyeon	4.9 ± 0.1fgh	25.2 ± 1.6hij	74.8 ± 1.8de	1536.9 ± 132.6b	79.7 ± 1.4ef	1562.2 ± 109.1b
15	Sunredbutter	41.9 ± 1.9a	73.5 ± 6.5ef	344.8 ± 14.5a	594.1 ± 24.8gh	386.7 ± 10.4a	667.6 ± 25.5f
16	Hyeseonmanchudae	5.0 ± 0.5fgh	171.5 ± 5.9b	51.0 ± 5.8jk	438.1 ± 12.2h	56.0 ± 5.1ijk	609.5 ± 9.5f
17	Sambokhacheong	5.0 ± 0.5fgh	21.1 ± 0.8ij	103.2 ± 5.3c	539.6 ± 7.7h	108.2 ± 4.7d	560.7 ± 7.0fg
18	Chunhachujeokchima	25.9 ± 2.2b	90.4 ± 6.4de	123.6 ± 8.2b	521.6 ± 29.6h	149.5 ± 8.4b	612.1 ± 29.3f
19	Yelpungjeokchima	2.4 ± 0.1kl	72.6 ± 13.2f	51.6 ± 1.5ijk	994.5 ± 24.1ef	54.0 ± 1.1jk	1067.1 ± 26.4de
20	Superseonpung	1.8 ± 0.2l	213 ± 48.2a	9.9 ± 0.2m	3888.2 ± 586.1a	11.7 ± 0.7m	4101.2 ± 448.5a
21	Tomalin	4.2 ± 1.1ghij	119.1 ± 12.9c	68.6 ± 2.0ef	985.6 ± 183.8ef	72.8 ± 0.8fgh	1104.7 ± 98.3bcd
22	Cheongchima	5.0 ± 1.6fghi	11.6 ± 0.1j	60.1 ± 9.9ghij	211.5 ± 19.3i	65.1 ± 5.8hij	223.1 ± 4.9h

Values are mean  ±  standard deviation of biological triplicates. Different letters between rows indicate statistically significant differences at p  < 0 0.05

*S/No* sample number

The content of lactucin and lactucopicrin in bolting stage leaves of lettuce are higher compared to the mature stage lettuce in this study as well as in previous study results [3, 15, 41]. All the cultivars considered in this study had higher lactucin (1.7- to 118.3-fold) and lactucopicrin (1.7- to 392.7-fold) concentrations at the bolting stage compared to their mature stage counterparts. The highest and least changes in the content of SLs were recorded in “Superseongpung” and “Sunredbutter” cultivars, respectively. The higher content of SLs at bolting stage compared to the mature stage could be attributed to de novo synthesis in new leaves generated at the latter stage as well as due to altered SLs expression in old leaves. Moreover, the older leaf tissues could accumulate more SLs through time. Literature concerning the relation between leaf age of lettuce and SLs are elusive. A similar observation to our result was reported in cultivated sunflower [43]. In another study, the total SLs concentration progressively increased as flowers of *Arnica Montana* get matured from buds to fully opened flowers and further increased as the petals withered [44]. Sweeter taste and more crispy texture of lettuce are the favorable sensory attributes for consumers [45]. Hence, sensory properties such as bitterness in part contribute to the overall acceptability of lettuce. The high content of SLs found in mature leaves as indicated in this study and flowering heads of plants [46] could diminish consumer acceptance.

**Fig. 4:**
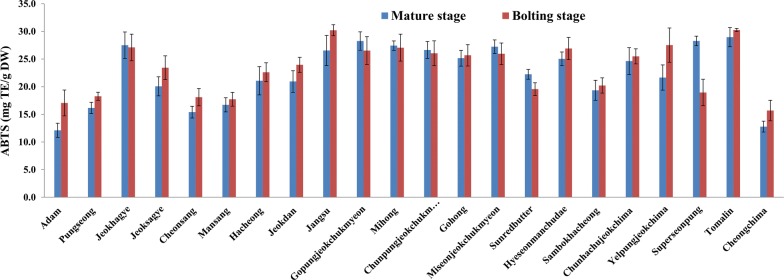
ABTS radical scavenging potentials of lettuce cultivars at mature and bolting stages

ABTS assay, a method based on scavenging of the stable cation radical ABTS·^+^, was used to estimate the antioxidant potential of methanol/water/formic acid extracts of lettuce. ABTS assay provides operational simplicity, diverse and flexible applicability in multiple media to determine both hydrophilic and lipophilic antioxidant potential of food extracts. In addition, as it presents absorbance peaks at 730 and 842 nm, ABTS avoid interferences that could arise from pigments in the lettuce extract or secondary reaction products between the chromogen and samples [47]. Significant differences in the content of phenolic compounds and antioxidant activity between green, green/red and red varieties were detected. Figure 4 presents the ABTS radical scavenging potentials of lettuce cultivars at mature and bolting stages. The ABTS radical scavenging potential ranged from 12.1 to 29.0 mg TE/g DW and 15.7 to 30.3 mg TE/g DW in mature stage and bolting stage lettuce leaves, respectively. The antioxidant activity of about 80% of the cultivars analyzed showed an increase (2.1 to 40.9%) in ABTS radical scavenging potential with maturity. Unlike other cultivars, the ABTS radical scavenging potential of a red commercial cultivar “Superseonpung” was approximately 50% lower at bolting stage compared to mature stage of lettuce. Studies concerning the effect of the maturity stage on the antioxidant potential of lettuce are elusive. However, in the case of other vegetables, such as spinach, the oxygen radical absorbance capacity (ORAC) values were found to be higher at mid-maturity stage compared to immature and mature leaves [48]. In another study of pac choi, spinach, red leaf lettuce, and romaine lettuce, mature head stage showed higher ORAC values than at the baby-sized stage [49]. Green/red and red pigmented lettuce cultivars were superior in their ABTS radical scavenging potential compared with green pigmented cultivars. This was supported by the significant correlation (Pearson correlation coefficient, R = 0.811, at *p* < 0.01) between the cyanidin content and ABTS radical scavenging activity demonstrating that cyanidin contributes to the antioxidant capacity of lettuce. The higher antioxidant activity in the red and green/red pigmented cultivars could also be related to their relatively higher amount of phenolic compounds. In concordance with the results of this study, Pérez-López et al. [6] observed that the hydrophilic antioxidant capacity increased together with anthocyanins for red pigmented lettuce.

### Multivariate analysis

**Fig. 5:**
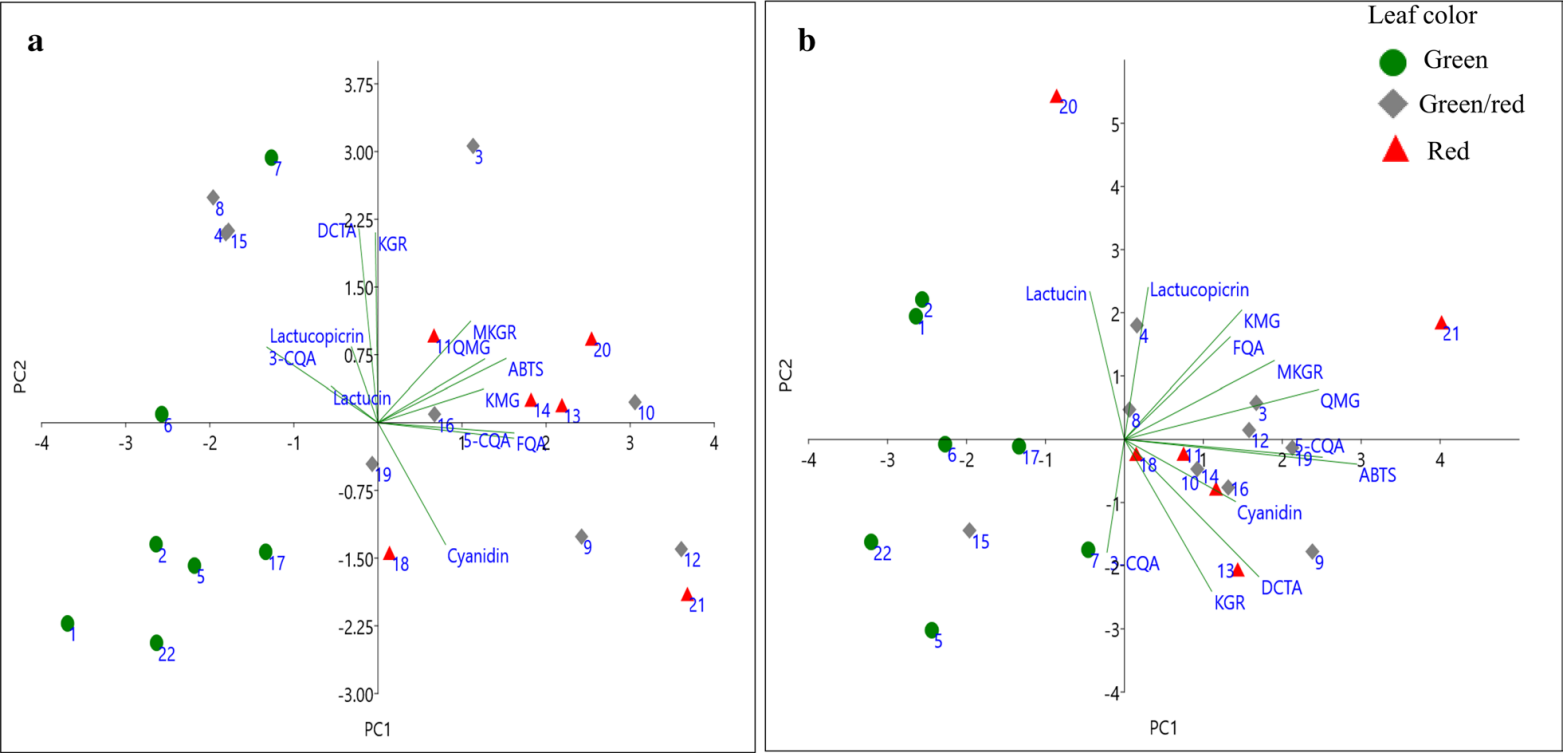
The loading and score plot of 22 lettuce cultivars at mature stage (**a**) and bolting stage (**b**). The score plot was grouped using leaf color (green, green/red, and red). The numerals (1–22) correspond to the cultivars described in Table 2

Principal components analysis (PCA) is a procedure for finding hypothetical variables that account for as much of the variance in multidimensional data set as possible [22]. In the PCA, a multivariate analysis was used in order to distinguish lettuce cultivars based on their leaf color (green, green/red, and red). Phenolic acids (3-CQA, 5-CQA, 5-FQA, DCTA), flavonoids (KGR, QMG, KMG, and MKGR), cyanidin, SLs (lactucin and lactucopicrin), and ABTS antioxidant activity data yielded three principal components with eigenvalues ≥ 1 of the correlation matrix accounting collectively for 84.1% and 78.0% of the total variance in the dataset of the mature and bolting stage lettuce cultivars, respectively (Additional file 1: Appendix S1). In mature lettuce cultivars, Eigen analysis of the loadings of the two significant principal components (PC1 and PC2) revealed that PC1 (X-axis, Fig. 5a) was mainly contributed by 5-FQA, 5-CQA, and ABTS. Meanwhile, PC2 (Y-axis, Fig. 5a) was mostly contributed by DCTA and KGR. The first three variables which contribute most to PC1 (X-axis, Fig. 5b) in mature leaf cultivars were 5-FQA, lactucin, and cyanidin whereas 5-CQA, ABTS, and MKGR contributed highly to PC2 (Y-axis, Fig. 5b). The score and loading plot (Fig. 5) was generated based on leaf color which were classified as green (7), green/red (9), and red (6) based on visual assessment at the field and laboratory. As can be seen in Fig. 5, the green pigmented lettuce cultivars were clearly grouped in the negative side of the X-axis (PC1) except cultivar “Hacheong” of the mature stage lettuce which was located at the upper left-hand quadrant of Fig. 5a. The isolation of “Hacheong” from other green pigmented cultivars of mature lettuce samples may be described by the significantly higher contents of DCTA and KGR compared with other green pigmented cultivars which are co-located in this region of the loading plot. All the red pigmented cultivars were located to the positive side of PC1 except the bolting stage leaves of “Superseonpung” cultivar which had significantly higher content of SLs compared with all other cultivars studied. The green/red pigmented cultivars of mature stage lettuce were significantly distributed in all quadrants with no prominent groupings. In contrast, green/red pigmented bolting stage cultivars were grouped to the positive side of PC1 except “Sunredbutter” which was co-located with green pigmented cultivars at the bottom left-hand side of the quadrant. The loading plot showed that the phenolic acids, flavonoids, and cyanidin are positively correlated with ABTS, indicating that the compounds contribute to the radical scavenging activity of lettuce extracts. Bolting stage leaf samples of the cultivars “Superseopung” and “Tomalin” were different from other cultivars because they have significantly higher content of SLs and QMG compared with others cultivars, respectively. These observations suggested that leaf color could partly contribute to the distinct phytoconstituents profile of lettuce such as phenolic compounds and antioxidant potential. However, a correlation between leaf color and the SLs content of lettuce was not observed.

## Conclusions

To summarize, we have identified, characterized and quantified the major phytochemicals (caffeoylquinic acids, dicaffeoylquinic acid, dicaffeoyltartaric acid, kaempferol conjugates, quercetin malonylglucoside, sesquiterpene lactones, and cyanidin) in 22 lettuce cultivars at mature and bolting stages using UPLC-PDA-Q-TOF-HDMS, UPLC, and HPLC. Their antioxidant potential was also explored using ABTS radical scavenging assay. The composition and contents of the studied metabolites and antioxidant activity varied significantly and was principally depend on leaf color, cultivar type, and maturity. The main phenolic acid components of lettuce were quinic and tartaric acid derivatives, whereas kaempferol derivatives were the dominant flavonoids across the entire sample. Bolting stage lettuce leaves accumulate relatively high amount of sesquiterpene lactones and some phenolic compounds such as QMG, MGKR, KMG, and 3-CQA compared to mature stage. Red and green/red lettuces contain higher MGKR compared to green pigmented cultivars in bolting stage lettuce leaves. Red pigmented lettuce also exhibited higher antioxidant capacity compared to the green and green/red pigmented lettuce, suggesting that cyanidin contributed to the antioxidant potential of lettuce leaf. The red pigmented lettuce showed higher content of cyanidin proportional to the intensity of the pigment. However, the green pigmented lettuce lack detectable amount of cyanidin. The PCA, where the score and loading plots were generated according to leaf color of lettuce cultivars, showed a prominent grouping of green pigmented lettuce cultivars in the negative side of the X-axis (PC1) except cultivar “Hacheong” of the mature stage lettuce. All the red pigmented cultivars were located to the positive side of PC1 except the bolting stage leaf sample of “Superseonpung” cultivar which had significantly higher content of SLs. The green/red pigmented cultivars of the mature stage lettuce were significantly distributed in all quadrants with no prominent groupings. Overall, higher amount of phytoconstituents were found to be accumulated in the red pigmented lettuce leaves compared to the green lettuce leaves. In addition, the contents of most of the metabolites in lettuce seem to increase with age of the leaves. Due to the bitter nature of SLs, their presence in significantly high amount in lettuce leaves at bolting stage could diminish consumer acceptance. However, alternatively, these leaves could be utilized by nutraceutical companies working to recover these compounds (Additional file 2: Appendix S2).

## Additional files

**Additional file 1: Appendix S1.** Loadings for the first three principal components (PC) of phenolic acids (3-CQA, 5-CQA, FQA, DCTA), flavonoids (KGR, QMG, KMG, and MKGR), cyanidin, SLs (lactucin and lactucopicrin), and antioxidant activity (ABTS).

**Additional file 2: Appendix S2.** Proposed chemical structures of identified compounds in the leaves of lettuce.

## References

[1] Kim DE, Shang X, Assefa AD (2018). Metabolite profiling of green, green/red, and red lettuce cultivars: variation in health beneficial compounds and antioxidant potential. Food Res Int.

[2] Touliatos D, Dodd IC, Mcainsh M (2016). Vertical farming increases lettuce yield per unit area compared to conventional horizontal hydroponics. Food Energy Secur.

[3] Seo MW, Yang DS, Kays SJ (2009). Sesquiterpene lactones and bitterness in korean leaf lettuce cultivars. HortScience.

[4] Gazula A, Kleinhenz MD, Scheerens JC, Ling PP (2007). Anthocyanin levels in nine lettuce (*Lactuca sativa*) cultivars: influence of planting date and relations among analytic, instrumented, and visual assessments of color. HortScience.

[5] Llorach R, Martínez-Sánchez A, Tomás-Barberán FA (2008). Characterisation of polyphenols and antioxidant properties of five lettuce varieties and escarole. Food Chem.

[6] Pérez-López U, Pinzino C, Quartacci MF (2014). Phenolic composition and related antioxidant properties in differently colored lettuces: a study by Electron Paramagnetic Resonance (EPR) kinetics. J Agric Food Chem.

[7] Ribas-Agustí A, Gratacós-Cubarsí M, Sárraga C (2011). Analysis of eleven phenolic compounds including novel p-coumaroyl derivatives in lettuce (*Lactuca sativa* L.) by Ultra-high-performance Liquid Chromatography with photodiode array and mass spectrometry detection. Phytochem Anal.

[8] Lopez A, Javier G-A, Fenoll J (2014). Chemical composition and antioxidant capacity of lettuce: comparative study of regular-sized (Romaine) and baby-sized (Little Gem and Mini Romaine) types. J Food Compos Anal.

[9] Tamaki H, Robinson RW, Anderson JL, Stoewsand GS (1995). Sesquiterpene lactones in virus-resistant lettuce. J Agric Food Chem.

[10] Chon SU, Jang HG, Kim DK (2005). Allelopathic potential in lettuce (*Lactuca sativa* L.) plants. Sci Hortic (Amsterdam).

[11] Pernice R, Scuderi D, Napolitano A (2007). Polyphenol composition and qualitative characteristics of fresh-cut lettuce in relation to cultivar, mulching, and storage. J Hortic Sci Biotechnol.

[12] Ferreres F, Gil MI, Castan M, Tomas-Barberan FA (1997). Phenolic metabolites in red pigmented lettuce (*Lactuca sativa*). Changes with minimal processing and cold storage. J Agric Food Chem.

[13] Bunning ML, Kendall PA, Stone MB (2010). Effects of seasonal variation on sensory properties and total phenolic content of 5 lettuce cultivars. J Food Sci.

[14] Gazula A, Kleinhenz MD, Streeter JG, Miller AR (2005). Temperature and cultivar effects on anthocyanin and chlorophyll b concentrations in three related lollo rosso lettuce cultivars. HortScience.

[15] Arakawa K, Minami M, Nakamura K (2009). Differences of sesquiterpene lactones content in different leaf parts and head formation stages in lettuce. Hortic Res.

[16] Yang X, Wei S, Liu B (2018). A novel integrated non-targeted metabolomic analysis reveals signi fi cant metabolite variations between different lettuce (*Lactuca sativa* L.) varieties. Hortic Res.

[17] Viacava GE, Gonzalez-Aguilar G, Roura SI (2014). Determination of phytochemicals and antioxidant activity in butterhead lettuce related to leaf age and position. J Food Biochem.

[18] Marin A, Ferreres F, Barbera GG, Gil MI (2015). Weather variability influences color and phenolic content of pigmented baby leaf lettuces throughout the season. J Agric Food Chem.

[19] Santos J, Oliveira MBPP, Ibanez E, Herrero M (2014). Phenolic profile evolution of different ready-to-eat baby-leaf vegetables during storage. J Chromatogr A.

[20] Price KR, DuPont MS, Shepherd R (1990). Relationship between the chemical and sensory properties of exotic salad crops-coloured lettuce (*Lactuca sativa*) and chicory (*Cichorium intybus*). J Sci Food Agric.

[21] Re R, Pellegrini N, Proteggente A (1999). Antioxidant activity applying an improved ABTS radical cation decolorization assay. Free Radic Biol Med.

[22] Hammer Ø, Harper DAT, Ryan PD (2001). PAST: paleontological statistics software package for education and data analysis. Palaeontol Electron.

[23] Assefa AD, Keum YS (2017). Effect of extraction solvent and various drying methods on polyphenol content and antioxidant activities of yuzu (*Citrus junos Sieb* ex Tanaka). J Food Meas Charact.

[24] Viacava GE, Roura SI, Berrueta LA (2017). Characterization of phenolic compounds in green and red oak-leaf lettuce cultivars by UHPLC-DAD-ESI-QToF/MS using MS^E^ scan mode. J Mass Spectrom.

[25] Hausler M, Ganzera M, Abel G (2002). Determination of caffeoylquinic acids and flavonoids in *Cynara scolymus* L. by High Performance Liquid Chromatography. Chromatographia.

[26] Schutz K, Kammerer D, Carle R, Schieber A (2004). Identification and quantification of caffeoylquinic acids and flavonoids from artichoke (*Cynara scolymus* L.) heads, juice, and pomace by HPLC-DAD-ESI/MSn. J Agric Food Chem.

[27] Clifford MN, Zheng W, Kuhnert N (2006). Profiling the chlorogenic acids of aster by HPLC-MSn. Phytochem Anal.

[28] Lin long Z, Harnly JM (2008). Identification of hydroxycinnamoylquinic acids of arnica flowers and burdock roots using a standardized LC-DAD-ESI/MS profiling method. J Agric Food Chem.

[29] Jaiswal R, Sovdat T, Vivan F, Kuhnert N (2010). Profiling and characterization by LC-MS^n^ of the chlorogenic acids and hydroxycinnamoylshikimate esters in mate (*Ilex paraguariensis*). J Agric Food Chem.

[30] Clifford MN, Johnston KL, Knight S, Kuhnert N (2003). Hierarchical scheme for LC-MS^n^ identification of chlorogenic acids. J Agric Food Chem.

[31] Pepe G, Sommella E, Manfra M (2015). Evaluation of anti-inflammatory activity and fast UHPLC-DAD-IT-TOF profiling of polyphenolic compounds extracted from green lettuce (*Lactuca sativa* L.; Var. Maravilla de Verano). Food Chem.

[32] Heimler D, Isolani L, Vignolini P (2007). Polyphenol content and antioxidative activity in some species of freshly consumed salads. J Agric Food Chem.

[33] Lech K, Witko K, Jarosz M (2014). HPLC-UV-ESI MS/MS identification of the color constituents of sawwort (*Serratula tinctoria* L.). Anal Bioanal Chem.

[34] Abu-reidah IM, Contreras MM, Arráez-román D (2013). Reversed-phase ultra-high-performance liquid chromatography coupled to electrospray ionization-quadrupole-time-of-flight mass spectrometry as a powerful tool for metabolic profiling of vegetables: *Lactuca sativa* as an example of its application. J Chromatogr A.

[35] Dupont MS, Mondin Z, Williamson G, Price KR (2000). Effect of variety, processing, and storage on the flavonoid glycoside content and composition of lettuce and endive. J Agric Food Chem.

[36] Chon SU, Boo HO, Heo BG, Gorinstein S (2012). Anthocyanin content and the activities of polyphenol oxidase, peroxidase and phenylalanine ammonia-lyase in lettuce cultivars. Int J Food Sci Nutr.

[37] Wu X, Gu L, Prior RL (2004). Characterization of anthocyanins and proanthocyanidins in some cultivars of Ribes, Aronia, and Sambucus and their antioxidant capacity. J Agric Food Chem.

[38] De Pascual-Teresa S, Sanchez-Ballesta MT (2008). Anthocyanins: from plant to health. Phytochem Rev.

[39] Neill SO, Gould KS (2003). Anthocyanins in leaves: light attenuators or antioxidants?. Funct Plant Biol.

[40] Graziani G, Ferracane R, Sambo P (2015). Profiling chicory sesquiterpene lactones by high resolution mass spectrometry. Food Res Int.

[41] Beharav A, Ben-David R, Malarz J (2010). Variation of sesquiterpene lactones in *Lactuca aculeata* natural populations from Israel, Jordan and Turkey. Biochem Syst Ecol.

[42] Ferioli F, Manco MA, Antuono LFD (2015). Variation of sesquiterpene lactones and phenolics in chicory and endive germplasm. J Food Compos Anal.

[43] Chou JC, Mullin CA (1993). Phenologic and tissue distribution of sesquiterpene lactones in cultivated sunflower (*Helianthus annuus* L.). J Plant Physiol.

[44] Douglas JA, Smallfield BM, Burgess EJ (2004). Sesquiterpene lactones in *Arnica montana*: a rapid analytical method and the effects of flower maturity and simulated mechanical harvesting on quality and yield. Planta Med.

[45] Pollard J, Kirk SFL, Cade JE (2002). Factors affecting food choice in relation to fruit and vegetable intake: a review. Nutr Res Rev.

[46] Chadwick M, Trewin H, Gawthrop F, Wagstaff C (2013). Sesquiterpenoids lactones: benefits to plants and people. Int J Mol Sci.

[47] Arnao MB (2000). Some methodological problems in the determination of antioxidant activity using chromogen radicals: a practical case. Trends Food Sci Technol.

[48] Pandjaitan N, Howard LR, Morelock T, Gil MI (2005). Antioxidant capacity and phenolic content of spinach as affected by genetics and maturation. J Agric Food Chem.

[49] Zhao X, Iwamoto T, Carey EE (2007). Antioxidant capacity of leafy vegetables as affected by high tunnel environment, fertilisation and growth stage. J Sci Agric.

