# Supplementary material for: Identification and quantification of selected metabolites in differently pigmented leaves of lettuce (Lactuca sativa L.) cultivars harvested at mature and bolting stages
Source: BMC Chem. 2019 Apr 19;13(1):56. doi: 10.1186/s13065-019-0570-2 (PMC6661726; doi:10.1186/s13065-019-0570-2)
Supplement: Supplementary file 1 — Additional file 1: Appendix S1. Loadings for the first three principal components (PC) of phenolic acids (3-CQA, 5-CQA, FQA, DCTA), flavonoids (KGR, QMG, KMG, and MKGR), cyanidin, SLs (lactucin and lactucopicrin), and antioxidant activity (ABTS). [file 13065_2019_570_MOESM1_ESM.docx]

**Appendix S1. Loadings for the first three principal components (PC) of phenolic acids (3-CQA, 5-CQA, FQA, DCTA), flavonoids (KGR, QMG, KMG, and MKGR), cyanidin, SLs (lactucin and lactucopicrin), and antioxidant activity (ABTS)**

| S/No | Variables | Mature stage | | |  |  | Bolting stage | | |
| --- | --- | --- | --- | --- | --- | --- | --- | --- | --- |
|  |  | PC1 | PC2 | PC3 | | | PC1 | PC2 | PC3 |
| 1 | 3-CQA | -0.34333 | 0.21641 | -0.13624 | | | -0.03714 | -0.30182 | 0.5213 |
| 2 | 5-CQA | 0.41969 | -0.04445 | 0.066604 | | | 0.42345 | -0.04752 | -0.26827 |
| 3 | 5-FQA | 0.42059 | -0.02942 | 0.062176 | | | 0.22636 | 0.27318 | 0.17103 |
| 4 | DCTA | -0.05871 | 0.55726 | -0.12836 | | | 0.28752 | -0.36689 | 0.26727 |
| 5 | KGR | -0.00777 | 0.54538 | -0.17424 | | | 0.18678 | -0.40604 | 0.32473 |
| 6 | QMG | 0.33 | 0.18278 | 0.20225 | | | 0.41563 | 0.13314 | -0.1812 |
| 7 | KMG | 0.32606 | 0.096808 | 0.045908 | | | 0.25115 | 0.34486 | 0.15182 |
| 8 | MKGR | 0.28642 | 0.29133 | -0.23503 | | | 0.3208 | 0.21 | 0.30476 |
| 9 | Cyanidin | 0.20895 | -0.35014 | -0.02851 | | | 0.23779 | -0.16603 | -0.44305 |
| 10 | Lactucin | -0.14452 | 0.10421 | 0.65177 | | | -0.07411 | 0.39441 | 0.087786 |
| 11 | Lactucopicrin | -0.08197 | 0.21748 | 0.63053 | | | 0.050858 | 0.40534 | 0.31167 |
| 12 | ABTS | 0.39631 | 0.18422 | 0.069817 | | | 0.49715 | -0.066 | -0.01577 |
|  | Eigenvalue | 5.29112 | 2.9388 | 1.86681 | | | 3.79694 | 3.39354 | 2.17247 |
|  | Variance (%) | 44.093 | 27.49 | 15.557 | | | 31.641 | 28.28 | 18.104 |
