# Supplementary material for: Identification and quantification of selected metabolites in differently pigmented leaves of lettuce (Lactuca sativa L.) cultivars harvested at mature and bolting stages
Source: BMC Chem. 2019 Apr 19;13(1):56. doi: 10.1186/s13065-019-0570-2 (PMC6661726; doi:10.1186/s13065-019-0570-2)
Supplement: Supplementary file 2 — Additional file 2: Appendix S2. Proposed chemical structures of identified compounds in the leaves of lettuce. [file 13065_2019_570_MOESM2_ESM.pptx]

## Slide 1
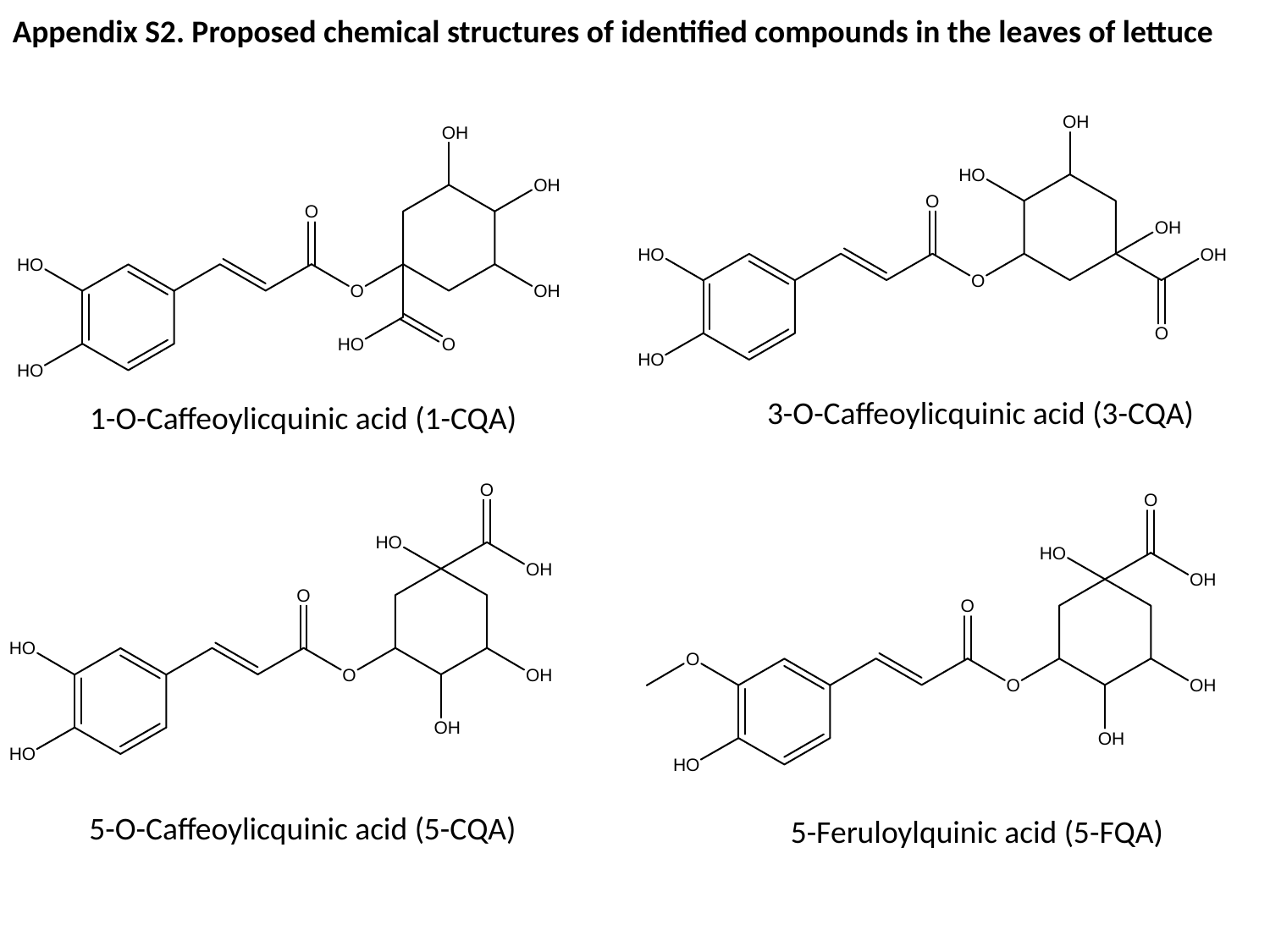

Appendix S2. Proposed chemical structures of identified compounds in the leaves of lettuce
3-O-Caffeoylicquinic acid (3-CQA)
1-O-Caffeoylicquinic acid (1-CQA)
5-O-Caffeoylicquinic acid (5-CQA)
5-Feruloylquinic acid (5-FQA)

## Slide 2
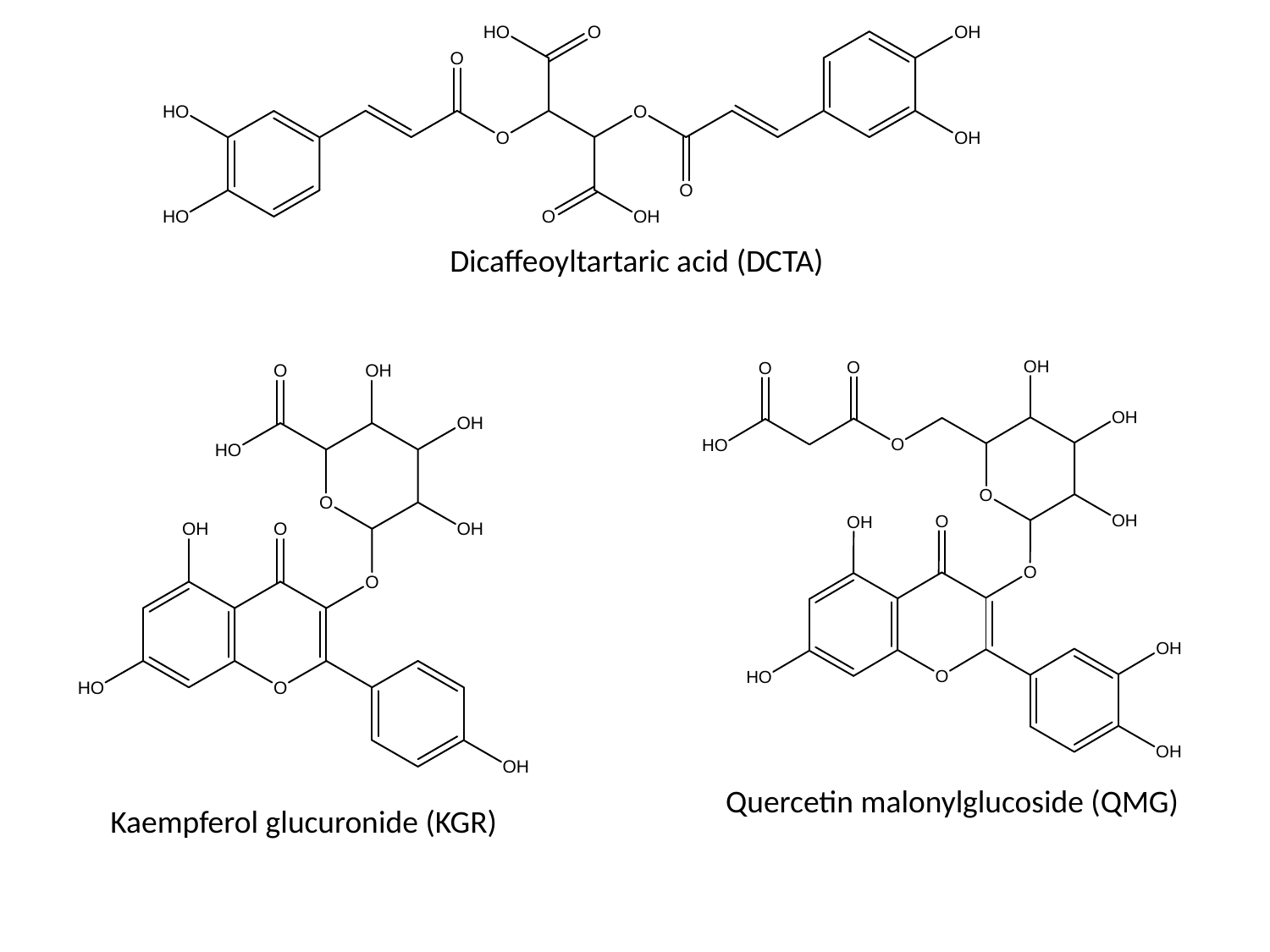

Dicaffeoyltartaric acid (DCTA)
Quercetin malonylglucoside (QMG)
Kaempferol glucuronide (KGR)

## Slide 3
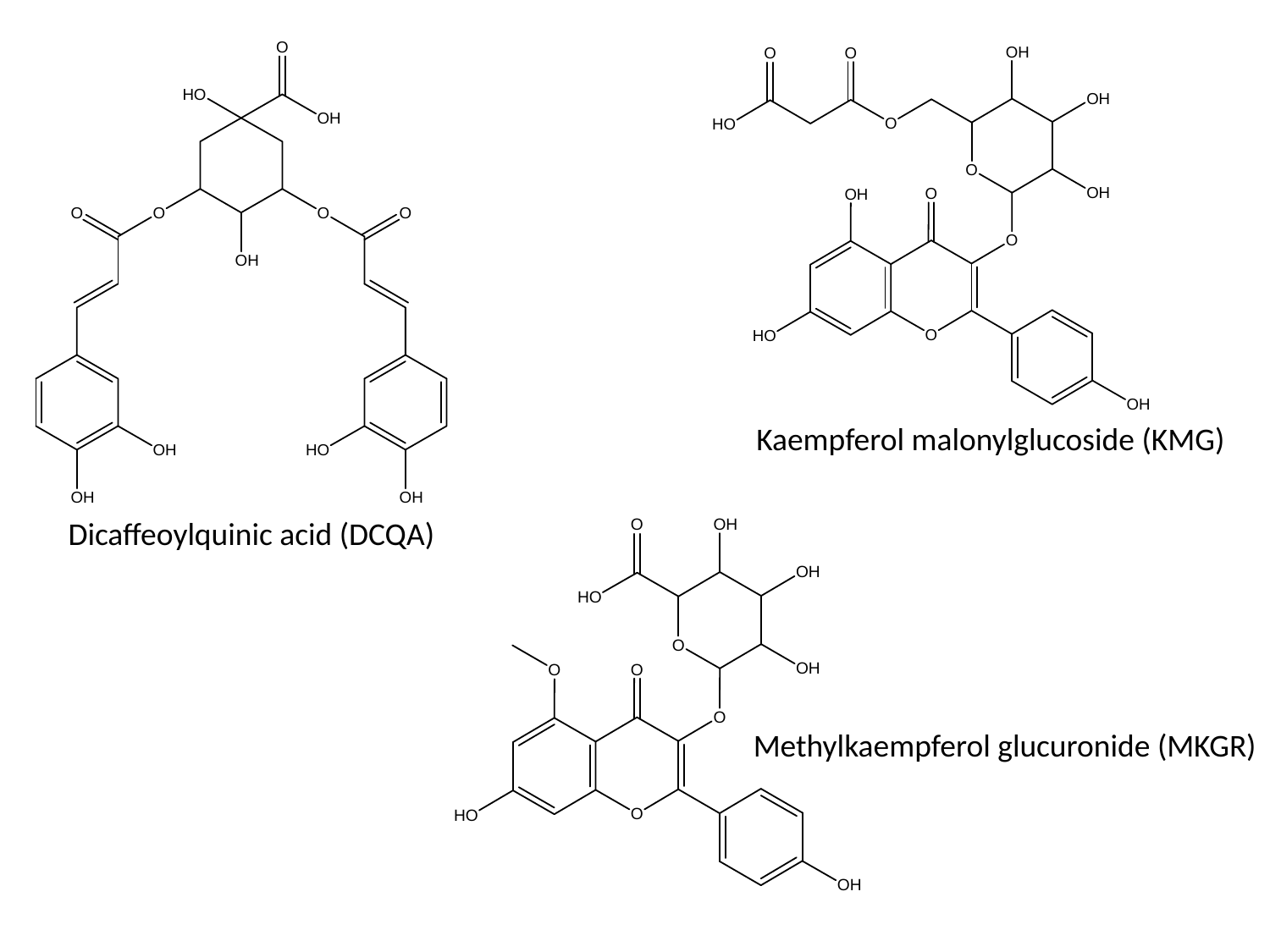

Kaempferol malonylglucoside (KMG)
Dicaffeoylquinic acid (DCQA)
Methylkaempferol glucuronide (MKGR)

## Slide 4
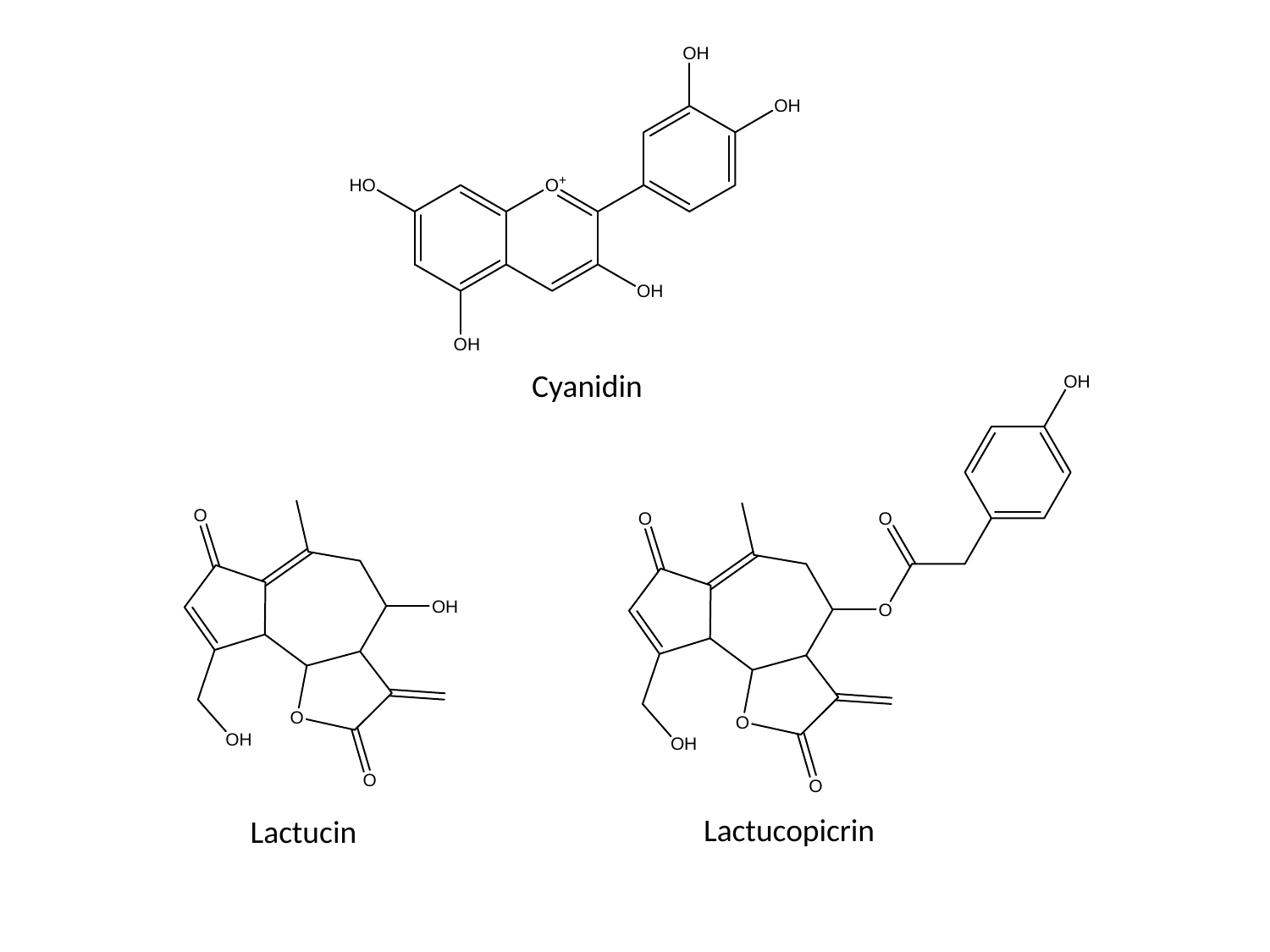

Cyanidin
Lactucopicrin
Lactucin
